# Prognostic value of the veterans affairs frailty index in older patients with non‐small cell lung cancer

**DOI:** 10.1002/cam4.4658

**Published:** 2022-03-26

**Authors:** David Cheng, Clark Dumontier, Ayesha R. Sheikh, Jennifer La, Mary T. Brophy, Nhan V. Do, Jane A. Driver, David P. Tuck, Nathanael R. Fillmore

**Affiliations:** ^1^ Massachusetts General Hospital Boston MA United States; ^2^ Department of Medicine Harvard Medical School Boston MA United States; ^3^ VA Boston Healthcare System Boston MA United States; ^4^ Brigham and Women's Hospital Boston MA United States; ^5^ Saint Vincent Hospital Worcester MA United States; ^6^ Boston University Boston MA United States; ^7^ Dana‐Farber Cancer Institute Boston MA United States

**Keywords:** electronic health records, frailty, non‐small cell lung, prognosis, veterans

## Abstract

**Background:**

Older patients with non‐small cell lung cancer (NSCLC) are a heterogeneous population with varying degrees of frailty. An electronic frailty index such as the Veterans Affairs Frailty Index (VA‐FI) can potentially help identify vulnerable patients at high risk of poor outcomes.

**Methods:**

NSCLC patients ≥65 years old and diagnosed in 2002–2017 were identified using the VA Central Cancer Registry. The VA‐FI was calculated using administrative codes from VA electronic health records data linked with Medicare and Medicaid data. We assessed associations between the VA‐FI and times to mortality, hospitalization, and emergency room (ER) visit following diagnosis by Kaplan–Meier analysis and multivariable stratified Cox models. We also evaluated the change in discrimination and calibration of reference prognostic models after adding VA‐FI.

**Results:**

We identified a cohort of 42,204 older NSCLC VA patients, in which 55.5% were classified as frail (VA‐FI >0.2). After adjustment, there was a strong association between VA‐FI and the risk of mortality (HR = 1.23 for an increase of four deficits or, equivalently, an increase of 0.129 on VA‐FI, *p* < 0.001), hospitalization (HR = 1.16 for four deficits, *p* < 0.001), and ER visit (HR = 1.18 for four deficits, *p* < 0.001). Adding VA‐FI to baseline prognostic models led to statistically significant improvements in time‐dependent area under curves and did not have a strong impact on calibration.

**Conclusion:**

Older NSCLC patients with higher VA‐FI have significantly elevated risks of mortality, hospitalizations, and ER visits following diagnosis. An electronic frailty index can serve as an accessible tool to identify patients with vulnerabilities to inform clinical care and research.

## Graphical abstract

Older non‐small cell lung cancer patients with higher values of an electronic frailty index have elevated risks of mortality and acute care utilization following diagnosis. Such an electronic index can help identify vulnerable patients to inform clinical decision‐making and research.

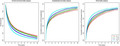

## 1 INTRODUCTION

Non‐small cell lung cancer (NSCLC) has a median age at diagnosis of 70 years and disproportionately affects older patients,^1^, ^2^ affecting a heterogeneous population that includes patients with varying functional status, cognitive impairment, and comorbid conditions.^3^, ^4^, ^5^ Limited evidence is available for guiding care and treatment of older cancer patients due to their under‐representation in clinical trials.^6^ Traditional assessments such as those based on chronological age and performance status may not account for the full range of vulnerabilities experienced by older patients with cancer.^7^ In the absence of assessments reflective of their full vulnerabilities, frail patients may be at risk of overtreatment with intensive therapies and fit older patients may be at risk of undertreatment with milder, less efficacious therapies.^8^, ^9^, ^10^

Current guidelines have recommended the adoption of geriatric assessments in older adults with cancer to help address this gap.^8^, ^9^, ^11^, ^12^ This has not been widely implemented in practice due to limitations in time, resources, and geriatrics expertise.^13^ Recently, electronic frailty indices have been developed to assess physiologic vulnerability based on the cumulative number of health deficits ascertained through electronic health record (EHR) and administrative data.^14^, ^15^ Electronic indices cannot fully capture elements of a geriatric assessment (e.g., gait speed). However, as these electronic indices depend only on data collected during routine care, they can be automatically computed and implemented at scale. This can facilitate automated screening of vulnerable patients^13^ for clinical care and also retrospective identification of frail patients for research, when alternative measures are not widely or consistently recorded. Some forms of a frailty index have already been developed for other settings in oncology, such as for patients who have breast cancer,^16^ ovarian cancer,^17^, ^18^ urologic malignancies,^19^ head‐and‐neck cancer,^20^, ^21^ multiple myeloma,^22^, ^23^, ^24^ and surgery.^25^ To our knowledge, no study has assessed electronic frailty indices in older NSCLC patients, outside of broad studies in overall cancer populations.^26^, ^27^

In this study, we measure frailty and assess its prognostic value by applying an electronic frailty index to older patients with NSCLC in the Veterans Affairs (VA) healthcare system. The VA Frailty Index (VA‐FI) was previously developed and validated in a large general cohort of older patients treated at the VA.^28^, ^46^ We evaluate both associations between the VA‐FI with mortality, hospitalizations, and emergency room (ER) visits in a large national cohort and its incremental value for predicting these outcomes when incorporated into prognostic models based on traditional predictors.

## 2 METHODS

### 2.1 Study population and data sources

We conducted a retrospective study primarily using EHR data from the VA Corporate Data Warehouse (CDW), which captures clinical, billing, and operational information for patients treated at VA facilities nationwide. Patients with lung cancer were identified as those with a record in the VA Central Cancer Registry (VACCR) in which “LUNG” was mentioned in the description of the primary tumor site. The VACCR records data on cancer patients from VA facilities nationwide and has been estimated to capture up to 90% of cases.^47^ Patients diagnosed with NSCLC were distinguished from those with small cell lung cancer through a manual review of lung cancer histology terms consistent with adenocarcinoma, squamous cell carcinoma, large cell carcinoma, and less common subtypes of NSCLC, as reported through ICD‐O (International Classification of Diseases for Oncology) codes in the VACCR. We focused on older patients who were at least 65 years old at the time of diagnosis, which enables incorporation of Medicare data for defining the VA‐FI among the population. The population was restricted to those diagnosed within 2002–2017 since the VACCR may have had underreporting of cancer cases in earlier years and Medicare data were available only through 2017. To limit attention to regular users of VA services, we excluded patients who did not have at least one record associated with an ICD code in each of the 3 years prior to diagnosis. We also excluded patients who failed to meet data quality control criteria, such as having the diagnosis date after last follow‐up date, having a stage 0 cancer diagnosis, or having an Eastern Cooperative Oncology Group (ECOG) score of 5 (death) at diagnosis. This study was conducted under a protocol approved by the VA Boston Healthcare System Research and Development Committee.

### 2.2 Baseline covariates

Patient demographics, including age, gender, and race/ethnicity were obtained from records in the CDW. Histological subtype was classified based on terms reported from ICD‐O codes available in the VACCR. The American Joint Committee on Cancer stage group, ECOG performance score, and sites of distant metastasis were ascertained based on records associated with the diagnosis from the VACCR. Performance scores reported as Karnofsky scores were converted to equivalent ECOG scores.^29^ Unknown performance scores were encoded as a separate category in the covariate for ECOG in the incremental value analyses. Data in the VACCR were abstracted from patient charts by cancer registrars at VA sites treating patients with cancer.^30^ The abstraction process complies with standards of the North American Association of Central Cancer Registries.^31^

### 2.3 VA frailty index (VA‐FI)

The VA‐FI for each patient was calculated as the proportion out of 31 equally weighted deficits (Table 1) incurred by the patient. These deficits account for impairments across multiple physiologic domains and were ascertained by the presence of specific sets diagnostic and procedure codes in patients' records. In particular, we extracted ICD9/10 (International Classification of Diseases Ninth/Tenth Revision), Current Procedural Terminology (CPT), and Healthcare Common Procedure Coding System codes within a 3‐year period prior to patients' NSCLC diagnosis date from the CDW and linked Medicare and Medicaid data to account for encounters both inside and outside the VA, though Medicaid data were available only through 2014. An updated version of the VA‐FI based on translating ICD9 codes used to define deficits in a previous version^28^ through the Centers for Medicare and Medicaid Services General Equivalence Mappings^32^ and manual review was applied to account for ICD10 codes incurred after October 2015.^46^ Patients were classified to be non‐frail (0–0.1), pre‐frail (0.1–0.2), mildly frail (0.2–0.3), and moderate‐to‐severely frail (>0.3), using previously published frailty index cutoffs.^33^, ^34^, ^35^

**TABLE 1:** Count and proportion of patients having each deficit that contributes to the VA‐FI, by frailty status: 0.‐0.1 (non‐frail), 0.1–0.2 (pre‐frail), 0.2–0.3 (mild frail), >0.3 (mod+ frail)

Deficit (%)	Non‐Frail (0–0.1)	Pre‐Frail (0.1–0.2)	Mild Frail (0.2–0.3)	Mod+ Frail (>0.3)
*n*	5440	13,321	11,730	11,713
Atrial fibrillation	119 (2.2)	978 (7.3)	2038 (17.4)	4005 (34.2)
Anemia	293 (5.4)	2316 (17.4)	4087 (34.8)	7227 (61.7)
Anxiety	148 (2.7)	1159 (8.7)	1785 (15.2)	3341 (28.5)
Arthritis	1107 (20.3)	5268 (39.5)	6386 (54.4)	8131 (69.4)
Coronary artery disease	522 (9.6)	4073 (30.6)	6042 (51.5)	8566 (73.1)
Cancer	1313 (24.1)	5772 (43.3)	6595 (56.2)	7784 (66.5)
Chronic pain	199 (3.7)	1395 (10.5)	2215 (18.9)	4054 (34.6)
Cerebrovascular disease	203 (3.7)	1662 (12.5)	3005 (25.6)	5542 (47.3)
Dementia	74 (1.4)	548 (4.1)	1196 (10.2)	3626 (31.0)
Depression	361 (6.6)	2050 (15.4)	3092 (26.4)	5368 (45.8)
Diabetes	597 (11.0)	3553 (26.7)	4821 (41.1)	6703 (57.2)
Durable medical equipment	160 (2.9)	967 (7.3)	1747 (14.9)	3638 (31.1)
Falls	48 (0.9)	439 (3.3)	952 (8.1)	2856 (24.4)
Fatigue	90 (1.7)	878 (6.6)	1795 (15.3)	4847 (41.4)
Failure to thrive	4 (0.1)	39 (0.3)	109 (0.9)	521 (4.4)
Gait abnormality	44 (0.8)	512 (3.8)	1528 (13.0)	4975 (42.5)
Hearing impairment/loss	693 (12.7)	3276 (24.6)	3977 (33.9)	5152 (44.0)
Heart failure	48 (0.9)	901 (6.8)	2347 (20.0)	5448 (46.5)
Hypertension	3196 (58.8)	10,908 (81.9)	10,698 (91.2)	11,280 (96.3)
Incontinence	34 (0.6)	331 (2.5)	614 (5.2)	1631 (13.9)
Kidney disease	75 (1.4)	1071 (8.0)	2307 (19.7)	4674 (39.9)
Liver disease or cirrhosis	129 (2.4)	963 (7.2)	1504 (12.8)	2590 (22.1)
Lung disease (COPD, asthma)	2148 (39.5)	8044 (60.4)	8399 (71.6)	9771 (83.4)
Muscular issues	39 (0.7)	551 (4.1)	1450 (12.4)	4781 (40.8)
Osteoporosis	66 (1.2)	427 (3.2)	691 (5.9)	1487 (12.7)
Parkinson's disease	27 (0.5)	192 (1.4)	332 (2.8)	829 (7.1)
Peripheral neuropathy	25 (0.5)	411 (3.1)	1083 (9.2)	3012 (25.7)
Peripheral vascular disease	462 (8.5)	3381 (25.4)	5080 (43.3)	7622 (65.1)
Thyroid disease	157 (2.9)	945 (7.1)	1426 (12.2)	2536 (21.7)
Vision comorbidity	540 (9.9)	2910 (21.8)	3705 (31.6)	5091 (43.5)
Weight loss	225 (4.1)	1258 (9.4)	1770 (15.1)	2705 (23.1)

*Note*. A patient is considered to incur a deficit if they have a record in CDW associated with the corresponding diagnosis and procedure codes, within 3 years prior to their diagnosis. The ICD9 codes associated with each deficit can be found in.^28^ An additional set of ICD10 codes are also used based on translating the ICD9 codes and based on manual review of ICD10 and CPT codes. Each patient can have at most one of each of the 31 deficit types.

### 2.4 Mortality, hospitalizations, and ER visits

The primary outcome of the study was all‐cause mortality as captured in the Veterans Health Administration Vital Status File, which aggregates mortality information captured from official sources, including reports from VA facilities, death certificates, VA National Cemetery Administration, Medicare, and the Social Security Administration data, among other sources.^36^ We considered acute hospitalizations and ER visits at VA facilities as secondary outcomes. Episodes of acute hospitalization visits were defined based on classifying the specialty of inpatient visits using a previously published approach.^37^ Specialties related to surgery or other scheduled services, such as urology, orthopedics, transplantation, anesthesiology, podiatry, and vascular, were excluded to focus on unplanned episodes of acute hospitalization. ER visits were ascertained by identifying outpatient visits with associated ER decision support system identifiers. The follow‐up was defined to be time from diagnosis to either death or last date of either a billable inpatient or outpatient encounter or a record in the fee treatment table for non‐VA care paid by the VA. For non‐censored patients, time to mortality, hospitalization, or ER visit was defined by the difference between the date of event and date of diagnosis.

### 2.5 Statistical analysis

The unadjusted survival for time from diagnosis to mortality and cumulative incidence of the first acute hospitalization and ER visit following diagnosis were estimated by Kaplan–Meier curves, stratifying by frailty status and also by ECOG score or stage. Stratified Cox proportional hazards models^38^ for time to mortality, first hospitalization, and first ER visit were fit with VA‐FI as a continuous covariate, adjusting for age at diagnosis, gender, race/ethnicity, and sites of distant metastasis as covariates and histological subtype and stage as stratification factors. The incremental value of incorporating VA‐FI into prognostic models was assessed by comparing the discrimination and calibration among four sets of models: (1) Stratified Cox models described above excluding the VA‐FI (“Baseline” model), (2) The Baseline model adding ECOG as a covariate (“Baseline with ECOG”), (3) The Baseline model adding VA‐FI as a covariate (“Baseline with VA‐FI”), and (4) The Baseline model adding both ECOG and VA‐FI as covariates (“Baseline with both ECOG and VA‐FI”). Models (1) and (2) serve as references for estimating the incremental value when adding VA‐FI to a model with traditional predictors and for assessing the relative incremental value compared to adding ECOG. Model (4) allows for assessment of incremental value when adding VA‐FI to models with ECOG. The cumulative risk of mortality, hospitalization, and ER visits were estimated from these models,^38^ for a range of short‐ and long‐term time points (1‐month, 1‐year, 3 years, 5 years, and 10 years after diagnosis). The discrimination was assessed by estimating the time‐dependent cumulative/dynamic area under the curve (AUC) for each model and landmark time.^39^, ^40^ The calibration was assessed by plotting the cumulative risk at each landmark time as estimated by Kaplan–Meier against mean predicted risks, within deciles of the predicted risk, using Nam‐D'Agnostino test to test for significant discrepancies.^41^ As a sensitivity, the assessments of discrimination and calibration for 1‐year time point were repeated in the subset of patients with known ECOG status. For the discrimination and calibration, Cox models were trained in a training set comprised of a random sample of 80% of the study sample and validated in the remaining 20%.

## 3 RESULTS

### 3.1 Patient cohort and baseline characteristics

We identified 42,204 older NSCLC patients who met the inclusion/exclusion criteria of the study (Table 2). The median follow‐up time was 6.53 years following diagnosis, and 85.0% of patients died, 63.0% incurred an acute hospitalization, and 54.2% incurred an ER visit during follow‐up. The baseline characteristics are reported in Table 3. The majority of patients were male (98.5%) and White (73.1%), with a substantial proportion being African American (12.6%). The mean age at diagnosis was 74.1 years. The two most common histological subtypes were adenocarcinoma (47.7%) and squamous cell carcinoma (47.9%). Most patients did not have performance status documented (60.5%), and 27.5% of patients had good (0–1) scores. The high degree of missing data reflects the lack of routine assessment and documentation of performance status in oncology notes for patients treated outside of clinical trials. The cohort includes both early (39.2% stage I or II) and late (53.3% III or IV) stage patients. Most patients had no distant metastasis at diagnosis (69.7%). Among the study cohort, 55.5% of patients were classified as frail (VA‐FI >0.2), of which 27.8% had VA‐FI >0.3. The mean VA‐FI was 0.25 with a standard deviation (SD) of 0.13. The most prevalent deficits underlying the VA‐FI include cancer, hypertension, and lung disease (chronic obstructive pulmonary disease or asthma).

**TABLE 2:** Sample selection criteria for defining study cohort

Selection criteria	Patients
“LUNG” primary site in VACCR	230,349
Histological confirmation using ICD‐O codes from VACCR	184,259
Age 65 at diagnosis	88,234
Include patients with diagnosis within 2002–2017	52,665
Exclude patients without ICD code in each of 3 years prior to diagnosis	42,357
Time‐to‐event (last follow‐up or death) and diagnosis date available	42,347
Diagnosis date prior to last follow‐up date (including death)	42,295
Exclude precancerous patients with stage 0	42,238
Exclude patients with ECOG 5 at diagnosis	42,204

Abbreviations: ICD, International Classification of Diseases; ICD‐O, International Classification Of Disease For Oncology; CDW, Corporate Data Warehouse; VACCR, VA Central Cancer Registry; ECOG, Eastern Cooperative Oncology Group Performance Status.

**TABLE 3:** Baseline characteristics of NSCLC study cohort by frailty status: 0.‐0.1 (non‐frail), 0.1–0.2 (pre‐frail), 0.2–0.3 (mild frail), >0.3 (mod+ frail). NSCLC subtype was determined based on manual review of ICD‐O codes reported in VACCR

Characteristics	Non‐frail (0–0.1)	Pre‐Frail (0.1–0.2)	Mild Frail (0.2–0.3)	Mod+ Frail (>0.3)
Sample size (*n*)	5440	13,321	11,730	11,713
Age at Dx, mean (SD)	72.07 (5.48)	73.23 (5.89)	74.42 (6.32)	75.83 (6.76)
Male (%)	5365 (98.6)	13,159 (98.8)	11,571 (98.6)	11,484 (98.0)
Race/ethnicity (%)				
White	3943 (72.5)	9742 (73.1)	8564 (73.0)	8615 (73.6)
African American	634 (11.7)	1681 (12.6)	1497 (12.8)	1514 (12.9)
Asian American Pacific Islander	40 (0.7)	88 (0.7)	74 (0.6)	98 (0.8)
American Indian or Alaska Native	20 (0.4)	42 (0.3)	47 (0.4)	54 (0.5)
Hispanic	123 (2.3)	306 (2.3)	259 (2.2)	280 (2.4)
Unknown	680 (12.5)	1462 (11.0)	1289 (11.0)	1152 (9.8)
Subtype (%)				
Adenocarcinoma	2746 (50.5)	6439 (48.3)	5552 (47.3)	5415 (46.2)
Large cell	143 (2.6)	324 (2.4)	268 (2.3)	230 (2.0)
Other	120 (2.2)	265 (2.0)	241 (2.1)	232 (2.0)
Squamous	2431 (44.7)	6293 (47.2)	5669 (48.3)	5836 (49.8)
AJCC stage (%)				
I	1449 (26.6)	3998 (30.0)	3562 (30.4)	3754 (32.0)
II	523 (9.6)	1284 (9.6)	1050 (9.0)	948 (8.1)
III	1335 (24.5)	3049 (22.9)	2592 (22.1)	2459 (21.0)
IV	1842 (33.9)	4044 (30.4)	3624 (30.9)	3523 (30.1)
Unknown	291 (5.3)	946 (7.1)	902 (7.7)	1029 (8.8)
ECOG (%)				
ECOG 0	772 (14.2)	1696 (12.7)	1348 (11.5)	1063 (9.1)
ECOG 1	910 (16.7)	2236 (16.8)	1872 (16.0)	1688 (14.4)
ECOG 2	280 (5.1)	787 (5.9)	876 (7.5)	1092 (9.3)
ECOG 3	112 (2.1)	358 (2.7)	453 (3.9)	822 (7.0)
ECOG 4	17 (0.3)	50 (0.4)	87 (0.7)	161 (1.4)
Unknown	3349 (61.6)	8194 (61.5)	7094 (60.5)	6887 (58.8)
Distant metastasis (%)				
None	3661 (67.3)	9349 (70.2)	8187 (69.8)	8218 (70.2)
Bone	730 (13.4)	1511 (11.3)	1326 (11.3)	1261 (10.8)
Lung	590 (10.8)	1393 (10.5)	1246 (10.6)	1170 (10.0)
Lymph	289 (5.3)	597 (4.5)	489 (4.2)	409 (3.5)
CNS	366 (6.7)	732 (5.5)	559 (4.8)	437 (3.7)
Liver	337 (6.2)	750 (5.6)	673 (5.7)	728 (6.2)
Perito	13 (0.2)	42 (0.3)	30 (0.3)	27 (0.2)
Pleura	219 (4.0)	439 (3.3)	464 (4.0)	493 (4.2)
Skin	21 (0.4)	43 (0.3)	30 (0.3)	29 (0.2)
Other	507 (9.3)	1107 (8.3)	904 (7.7)	876 (7.5)

Abbreviations: AA, African American; AJCC American Joint Committee on Cancer; Dx, diagnosis; ECOG, Eastern Cooperative Oncology Group Performance Status.

### 3.2 Association of outcomes with frailty

**FIGURE 1:**
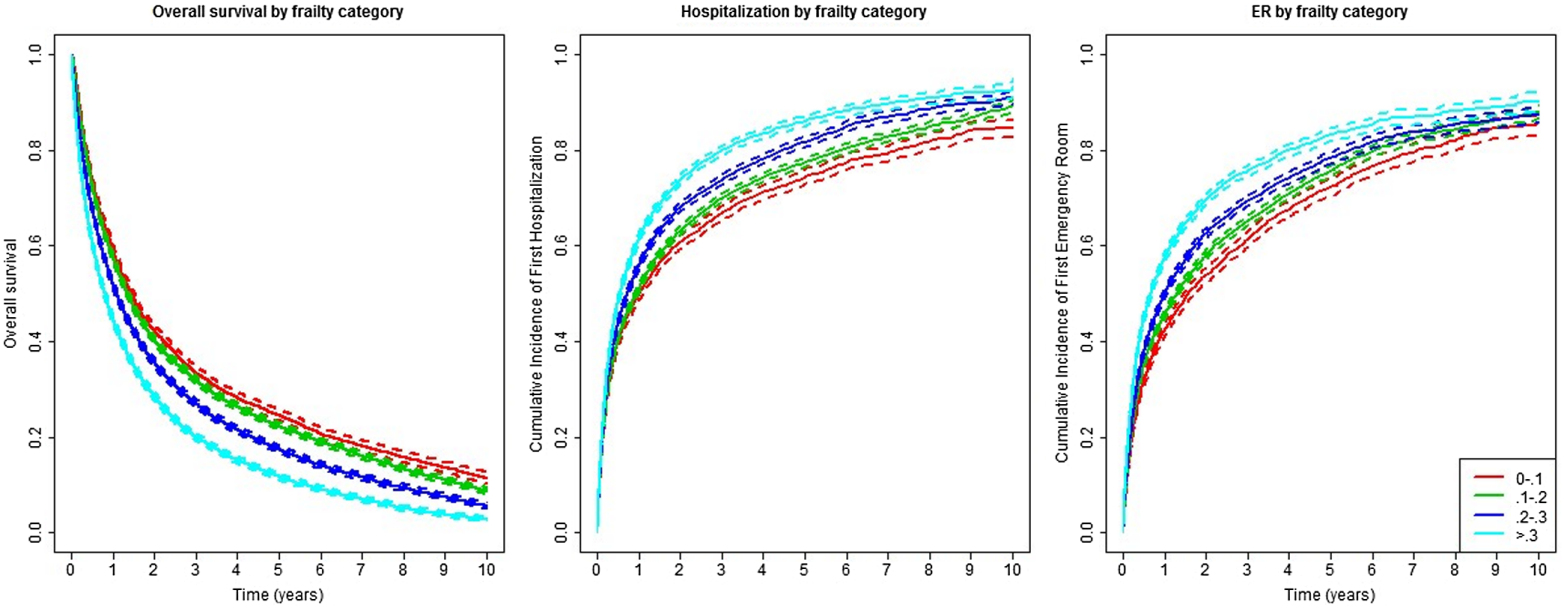
Kaplan–Meier estimates of the survival and cumulative incidence of acute hospitalizations and ER visits over years after diagnosis by frailty categories, among the study cohort. The frailty categories include non‐frail (0–0.1), pre‐frail (0.1–0.2), mild frail (0.2–0.3), and mod+ frail (>0.3). Dashed lines are pointwise 95% confidence intervals

**FIGURE 2:**
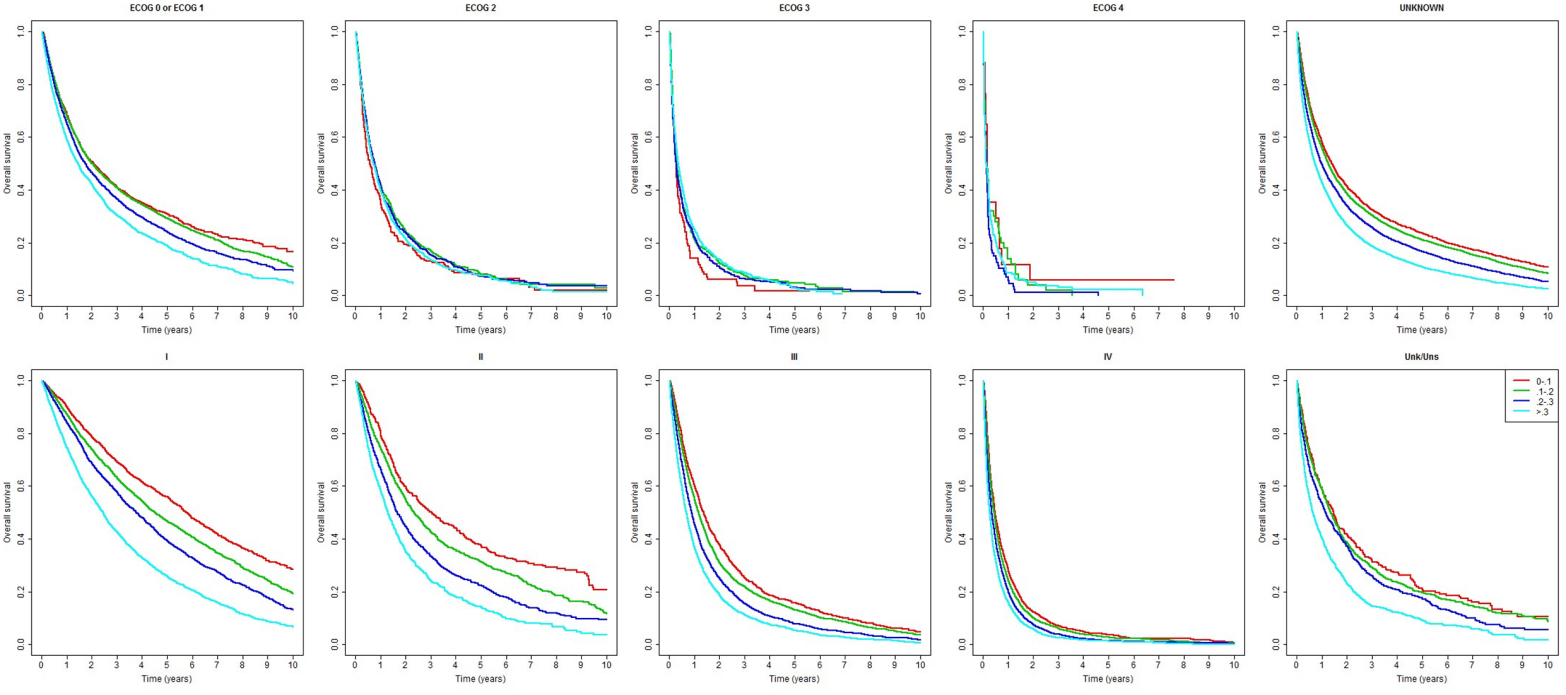
Kaplan–Meier estimates of the survival over years after diagnosis by frailty categories, among the study cohort, further stratified by ECOG performance status (ECOG 0 or 1, ECOG 2, ECOG 3, ECOG 4, and Unknown ECOG) and AJCC stage (I, II, III, IV, and Unknown) at diagnosis. Abbreviations: AJCC American Joint Committee on Cancer; ECOG, Eastern Cooperative Oncology Group Performance Status

Patients with higher VA‐FI at baseline had significantly lower survival and higher incidences of acute hospitalizations and ER visits (Figure 1). The 1‐year survival ranged from 59.3% (95% CI 58.0%–60.9%) for non‐frail patients to 44.7% (43.8%–45.6%) for moderate‐to‐severely frail patients, with similar differences seen for hospitalization and ER endpoints. The differences in survival persisted for as long as 10 years after diagnosis. The VA‐FI still differentiated risk for patients with 0–1 or unknown ECOG scores and patients with stage I‐III disease or unknown stage (Figure 2), although differentiation was weaker among those with higher ECOG scores and stage IV disease. Associations between VA‐FI with mortality and adverse outcomes held after adjustment for demographics and clinical features (Table 4). The magnitude of association is generally strong, with an accumulation of four additional deficits corresponding to a hazard ratio (HR) of 1.23 (95% CI 1.21–1.24; *p* < 0.001) for mortality, 1.16 (1.15–1.18; *p* < 0.001) for hospitalization, and 1.18 (1.17–1.20; *p* < 0.001) for ER visits. Having four additional deficits approximates a 1‐SD increase in the VA‐FI in the cohort (0.129 on the 0–1 scale) and is consistent with shifting from lower to higher frailty categories defined in Section 2.3.^28^, ^33^, ^34^, ^35^, ^46^

**TABLE 4:** Estimates from stratified Cox models for mortality, first acute hospitalization, and first ER visit after diagnosis, including the hazard ratio (HR), 95% confidence intervals for the HR (CI‐L, CI‐U), *z*‐statistic, and *p*‐value for the test that the conditional log HR is 0

Predictor	HR	CI‐L	CI‐U	*z*	*p*	HR	CI‐L	CI‐U	*z*	*p*	HR	CI‐L	CI‐U	*z*	*p*
Age at Dx (years)	1.025	1.023	1.027	29.089	<0.001	0.997	0.995	0.999	−2.599	0.009	0.995	0.992	0.997	−4.869	<0.001
Male	1.381	1.259	1.516	6.798	<0.001	1.261	1.137	1.399	4.397	<0.001	1.053	0.949	1.168	0.976	0.329
Race															
African American	0.962	0.932	0.993	−2.394	0.017	1.087	1.049	1.126	4.613	<0.001	1.164	1.121	1.208	7.897	<0.001
Asian American Pacific Islander	0.925	0.818	1.047	−1.227	0.220	0.835	0.720	0.969	−2.371	0.018	1.030	0.887	1.196	0.388	0.698
American Indian or Alaska Native	0.862	0.725	1.023	−1.700	0.089	1.075	0.896	1.291	0.776	0.438	1.196	0.992	1.441	1.876	0.061
Hispanic	0.971	0.904	1.043	−0.801	0.423	1.135	1.050	1.226	3.181	0.001	1.336	1.233	1.446	7.115	<0.001
Unknown	1.473	1.426	1.522	23.306	<0.001	0.950	0.910	0.991	−2.353	0.019	0.627	0.594	0.662	−17.019	<0.001
Site of metastasis															
Distant bone	1.482	1.429	1.537	21.102	<0.001	1.284	1.226	1.346	10.528	<0.001	1.238	1.175	1.304	8.000	<0.001
Distant lung	0.973	0.936	1.011	−1.400	0.161	0.991	0.944	1.040	−0.369	0.712	0.882	0.835	0.932	−4.480	<0.001
Distant lymph	1.157	1.101	1.216	5.749	<0.001	1.089	1.022	1.160	2.650	0.008	1.128	1.052	1.210	3.369	<0.001
Distant CNS	1.542	1.469	1.620	17.329	<0.001	1.377	1.294	1.465	10.110	<0.001	1.226	1.142	1.315	5.671	<0.001
Distant liver	1.296	1.247	1.347	13.106	<0.001	1.181	1.124	1.241	6.602	<0.001	1.125	1.064	1.188	4.177	<0.001
Distant perito	1.508	1.443	1.577	18.130	<0.001	1.206	1.137	1.278	6.290	<0.001	1.075	1.006	1.150	2.121	0.034
Distant Pleura	1.422	1.180	1.714	3.704	<0.001	0.967	0.744	1.259	−0.246	0.805	0.745	0.539	1.030	−1.780	0.075
Distant skin	1.419	1.345	1.498	12.727	<0.001	1.265	1.181	1.356	6.669	<0.001	1.288	1.194	1.389	6.539	<0.001
Distant other	1.258	1.053	1.504	2.522	0.012	1.253	1.004	1.565	1.993	0.046	1.146	0.886	1.481	1.039	0.299
eFI (increase of 0.129 or four deficits)	1.227	1.213	1.240	37.496	<0.001	1.161	1.147	1.176	23.336	<0.001	1.181	1.165	1.197	24.152	<0.001

*Note*. Patient follow‐up is censored by the date of last record in the CDW with an ICD code. The stratification factors are histological subtype and cancer stage. VA‐FI is entered as a continuous predictor in the Cox model.

Abbreviations: Dx, diagnosis; VA‐FI, VA Frailty Index.

### 3.3 Incremental value of VA‐FI

Adding VA‐FI to baseline prognostic models generally led to statistically significant improvements in time‐dependent AUCs (Table 5). For mortality, the Baseline model achieved AUCs ranging 0.76–0.82 for short‐ and long‐term time points for mortality, and adding the VA‐FI to the Baseline model significantly increased the AUC by 0.01–0.03. Adding the VA‐FI to the Baseline model with ECOG further significantly increased the AUC by 0.01–0.02. Similar patterns of improvement from adding the VA‐FI to the Baseline model were observed for hospitalizations and ER visits, with the most pronounced improvements occurring for long‐term time points (0.07 for 10 year hospitalizations 0.06 for 5‐year ER visits). Compared to the Baseline model with ECOG, models that additionally included the VA‐FI generally exhibited further improvements in AUC, with the exception of 5‐ and 10‐year hospitalizations. These patterns of improvement were also similar among patients with known ECOG, although the gains from adding VA‐FI to baseline was lower than that of adding ECOG in some cases (e.g., short‐term mortality outcomes) due to improved performance of models with ECOG (Table  S1).

**TABLE 5:** Cumulative/dynamic time‐dependent AUC and 95% CIs at landmark time points following diagnosis

	AUC (95% CI)	*p* (vs. Baseline)	*p* (vs. ECOG)	AUC (95% CI)	*p* (vs. Baseline)	*p* (vs. ECOG)	AUC (95% CI)	*p* (vs. Baseline)	*p* (vs. ECOG)
1‐month									
Baseline	0.76 (0.741–0.778)	‐	‐	0.643 (0.625–0.66)	‐	‐	0.635 (0.616–0.654)	‐	‐
Baseline+ECOG	0.77 (0.752–0.788)	<0.001	‐	0.649 (0.632–0.667)	<0.001	‐	0.656 (0.637–0.675)	<0.001	‐
Baseline+VAFI	0.775 (0.758–0.793)	<0.001	0.131	0.649 (0.632–0.667)	0.018	0.999	0.642 (0.623–0.66)	0.134	0.04
Baseline+ECOG+VAFI	0.785 (0.767–0.802)	<0.001	<0.001	0.655 (0.638–0.672)	<0.001	0.026	0.661 (0.642–0.679)	<0.001	0.123
1‐year									
Baseline	0.807 (0.798–0.816)	‐	‐	0.69 (0.677–0.703)	‐	‐	0.652 (0.638–0.666)	‐	‐
Baseline+ECOG	0.817 (0.808–0.826)	<0.001	‐	0.696 (0.683–0.709)	<0.001	‐	0.681 (0.668–0.694)	<0.001	‐
Baseline+VAFI	0.816 (0.807–0.825)	<0.001	0.459	0.71 (0.697–0.722)	<0.001	<0.001	0.68 (0.666–0.693)	<0.001	0.875
Baseline+ECOG+VAFI	0.824 (0.815–0.833)	<0.001	<0.001	0.713 (0.701–0.726)	<0.001	<0.001	0.698 (0.685–0.711)	<0.001	<0.001
3‐year									
Baseline	0.818 (0.808–0.828)	‐	‐	0.723 (0.708–0.739)	‐	‐	0.654 (0.636–0.673)	‐	‐
Baseline+ECOG	0.824 (0.815–0.834)	<0.001	‐	0.728 (0.713–0.744)	0.074	‐	0.677 (0.659–0.696)	0.01	‐
Baseline+VAFI	0.829 (0.82–0.839)	<0.001	0.009	0.746 (0.73–0.761)	<0.001	<0.001	0.689 (0.67–0.707)	<0.001	0.238
Baseline+ECOG+VAFI	0.833 (0.824–0.843)	<0.001	<0.001	0.748 (0.733–0.764)	<0.001	<0.001	0.7 (0.681–0.719)	<0.001	<0.001
5 year									
Baseline	0.813 (0.801–0.826)	‐	‐	0.728 (0.707–0.749)	‐	‐	0.639 (0.612–0.666)	‐	‐
Baseline+ECOG	0.817 (0.804–0.829)	0.136	‐	0.731 (0.71–0.752)	0.418	‐	0.689 (0.662–0.715)	<0.001	‐
Baseline+VAFI	0.828 (0.816–0.84)	<0.001	<0.001	0.766 (0.745–0.788)	<0.001	<0.001	0.7 (0.673–0.726)	<0.001	0.437
Baseline+ECOG+VAFI	0.828 (0.816–0.841)	<0.001	<0.001	0.765 (0.744–0.787)	<0.001	<0.001	0.721 (0.694–0.747)	<0.001	<0.001
10‐year									
Baseline	0.774 (0.743–0.805)	‐	‐	0.709 (0.651–0.767)	‐	‐	0.671 (0.609–0.734)	‐	‐
Baseline+ECOG	0.765 (0.734–0.795)	0.058	‐	0.693 (0.633–0.753)	0.147	‐	0.744 (0.69–0.798)	0.008	‐
Baseline+VAFI	0.8 (0.769–0.83)	<0.001	<0.001	0.775 (0.725–0.826)	<0.001	<0.001	0.719 (0.657–0.782)	0.1	0.396
Baseline+ECOG+VAFI	0.788 (0.757–0.819)	0.03	<0.001	0.756 (0.706–0.806)	0.001	<0.001	0.783 (0.727–0.839)	0.001	0.015

*Note*. Baseline refers to the baseline stratified Cox model for the hazard of each type of event using age, gender, and site of metastasis, stratifying by stage and histological subtype. “Baseline+ECOG” refers to the same model except that ECOG is added as a covariate, and similarly for “Baseline+VAFI” and “Baseline+ECOG+VAFI.” *p*‐values are based on tests for the null that the AUC of each model differs from that of the “Baseline” model or the “Baseline+ECOG” model. For each landmark time and outcome, pairwise comparisons of the time‐dependent AUCs are made with the Baseline (“vs baseline”) and Baseline+ECOG (“vs ecog”) models.

Abbreviations: ECOG, Eastern Cooperative Oncology Group Performance Status; VA‐FI, VA Frailty Index.

**FIGURE 3:**
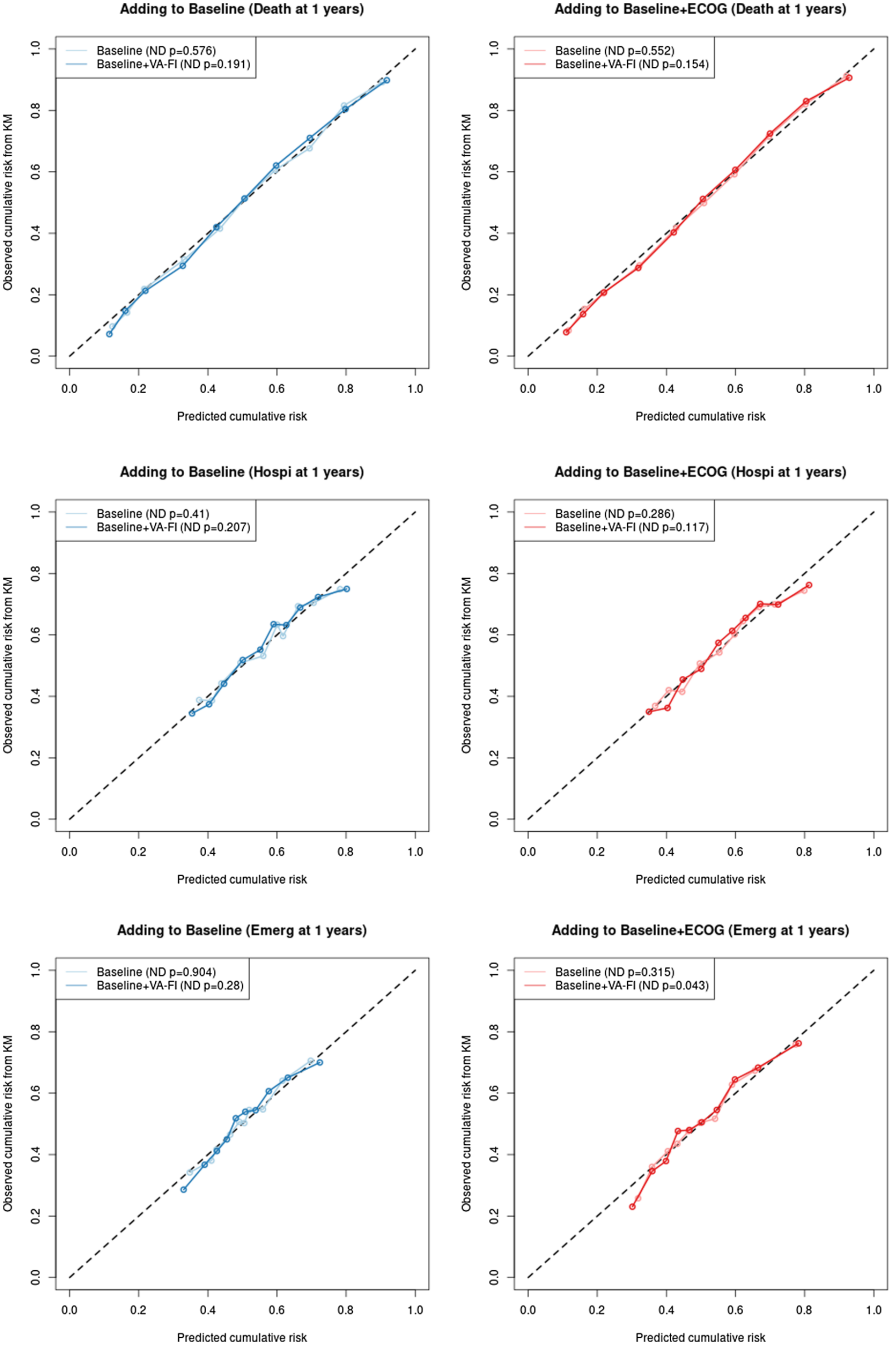
Calibration plot for predicted risk of mortality, hospitalization, and ER visit by 1 year after diagnosis, based on Baseline and Baseline+VAFI models (Blue) and Baseline+ECOG and Baseline+ECOG+VAFI models (Red). The calibration plots show the observed risk, as estimated by Kaplan–Meier, against the mean predicted risk in deciles of the predicted risks. The p‐values are from Nam‐D'Agnostino tests, which test against the null hypothesis that there are no differences between mean predicted and observed risks for each model. Abbreviations: ND, Nam‐D'Agnostino test; VA‐FI, VA Frailty Index; KM, Kaplan–Meier

Calibration plots exhibited a close degree of agreement between predicted and observed risks for early endpoints that was not reduced by addition of the VA‐FI (Figure 3). For mortality and hospitalizations, the predicted risks by 1‐year after diagnosis agreed closely with a wide range observed risks regardless of whether VA‐FI was included in the models. For ER visits, adding the VA‐FI to the Baseline model with ECOG did lead to marginally significant discrepancies using the Nam‐D'agnostino test, but the absolute differences were small and unlikely to be clinically significant. For later time points and sensitivity analyses among patients with known ECOG, significant discrepancies were generally detected regardless of whether VA‐FI was included (Figures S1 and S2).

## 4 DISCUSSION

Older patients with NSCLC constitute a large and heterogeneous population who are underrepresented in clinical research. This calls for novel approaches to characterize their risk of adverse outcomes. Electronic frailty indices such as the VA‐FI can be readily calculated from data collected during routine care to help evaluate vulnerability. Here, we applied the VA‐FI to a cohort of NSCLC patients at the VA and found that frailty was prevalent among VA patients with NSCLC according to the VA‐FI, with over half of the patients being classified to be at least mildly frail and over a quarter moderate‐to‐severely frail. These rates of frailty are either similar or higher to rates reported in other studies on frailty in solid tumors,^26^, ^27^, ^42^ which may be influenced by the greater burden of chronic conditions among Veterans.^43^ Frail patients were at elevated risk of mortality and adverse outcomes, even after adjustment for demographics and standard clinical predictors. This is consistent with studies in other cancers^22^, ^25^ and with smaller studies in NSCLC.^4^, ^44^ The strong associations suggest that such electronic frailty indices can identify vulnerable patients who are at increased risk of adverse outcomes.

Adding the VA‐FI to baseline prognostic models led to improvements in AUCs for discriminating both short‐ and long‐term adverse outcomes. These improvements are generally comparable or higher than improvements from adding ECOG. As ECOG was unknown for a majority of patients, the results on models with ECOG reflect the real‐world performance after accounting for its incomplete measurement. The impact of adding the VA‐FI to models among those with known ECOG status were similar, with some exceptions such as for short‐term mortality outcomes. In contrast to ECOG, the VA‐FI can be automatically computed using EHR and administrative data only, requiring no time from clinicians to perform an assessment. It can thus be broadly implemented in EHR systems to systematically screen for frailty among patients who are diagnosed with NSCLC to inform clinical care. The VA‐FI also provides a standardized measure of frailty that can be applied retrospectively to EHR and administrative data for research that would not be subject to incomplete documentation. The gains in AUC when adding VA‐FI to the baseline with ECOG model also suggest that VA‐FI captures complementary prognostic information that would refine estimates of risk, even when ECOG is included in the model. Given the low cost calculating the VA‐FI and the potential benefits to correct anticipation of adverse outcomes and early intervention, these refinements to prognostic models from incorporating the VA‐FI can potentially be clinically meaningful, though further work would be needed to evaluate the impact of adopting the VA‐FI in patient assessments on outcomes.

A major strength of this study is the ability to assess frailty among a large national cohort of older NSCLC patients. This enabled the evaluation of associations and prediction performance with VA‐FI in a broad NSCLC patient population. As curated data on traditional oncology assessments were available through the VACCR, we were able to adjust for clinical predictors including stage and ECOG score and compare the performance of prognostic models against reference models based on these standard predictors. Whereas many of the existing studies on frailty indices in cancer evaluate only associations with outcomes, this study also assessed the changes in discrimination and calibration to demonstrate the expected impact on out‐of‐sample risk prediction.

One limitation of our study was that the prognostic models did not incorporate information on treatments following diagnosis. This would allow for models to estimate predicted outcomes under different treatments and potentially improve the actionability of prediction results, though further work would be needed to account for confounding.^45^ Adjustment for treatment in models may either attenuate or inflate the associations between the VA‐FI and outcomes, depending on patterns of utilization and the safety and effectiveness of the treatment among frail and non‐frail patients. Additionally, hospitalizations and ER visits outside the VA are not captured in VA data. We did not utilize the Medicare and Medicaid data for the secondary outcomes, as definitions that were reliable and known to be consistent with definitions used in the CDW were not available. Nevertheless, the cumulative incidence of hospitalizations and ER visits were within the range of estimates from previous studies and do not appear to be severely underreported.^48^, ^49^ Associations with the VA‐FI could be attenuated if those with non‐VA hospitalizations or ER visits had lower VA‐FI scores than others in the cohort. Finally, the VA population differs from non‐VA populations in several respects, most notably in the high proportion of male patients. Additional studies would be needed to confirm the generalizability of our findings to other populations.

## 5 CONCLUSION

Among older patients with NSCLC, the VA‐FI is significantly associated with times to mortality, hospitalization, and ER visit following diagnosis. Incorporating the VA‐FI in prognostic models based on demographics and traditional clinical predictors led to significant improvements in discrimination. These results suggest that electronic indices such as the VA‐FI can help identify patients with vulnerabilities that put them at an elevated risk of experiencing adverse outcomes. Further research on how electronic frailty indices can help optimize treatment and care of NSCLC patients is warranted.

## CONFLICT OF INTEREST

No conflict of interest was disclosed.

## AUTHOR CONTRIBUTIONS

Conceptualization: DC, CM, ARS, JAD, DPT, and NRF; Data curation: DC, ARS, JL, and DPT; Formal analysis: DC and JL; Funding acquisition: NVD, MTB, and NRF; Investigation: DC, ARS, DPT, and NRF; Methodology: DC, DPT, and NRF; Project administration: DC and NRF; Resources: NVD, MTB, and NRF; Software: DC, JL, and NRF; Supervision: NVD, MTB, DPT, and NRF; Validation: DC and JL; Visualization: DC; Writing – original draft: DC, CM, DPT, and NRF; Writing – review and editing: DC, CM, JL, NVD, JAD, DPT, and NRF.

## ETHICS APPROVAL STATEMENT

This study was conducted under a protocol approved by the VA Boston Healthcare System Research and Development Committee.

## Supporting information


Figure S1

Figure S2

Table S1

## Data Availability

The VA CDW and Medicare data are not available to be publicly shared per VA policies.
