# Supplementary material for: Prognostic value of the veterans affairs frailty index in older patients with non‐small cell lung cancer
Source: Cancer Med. 2022 Mar 26;11(15):3009–22. doi: 10.1002/cam4.4658 (PMC9359868; doi:10.1002/cam4.4658)
Supplement: Supplementary file 1 — Figure S1 [file CAM4-11-3009-s003.docx]

# Calibration Plots at Additional Landmark Times


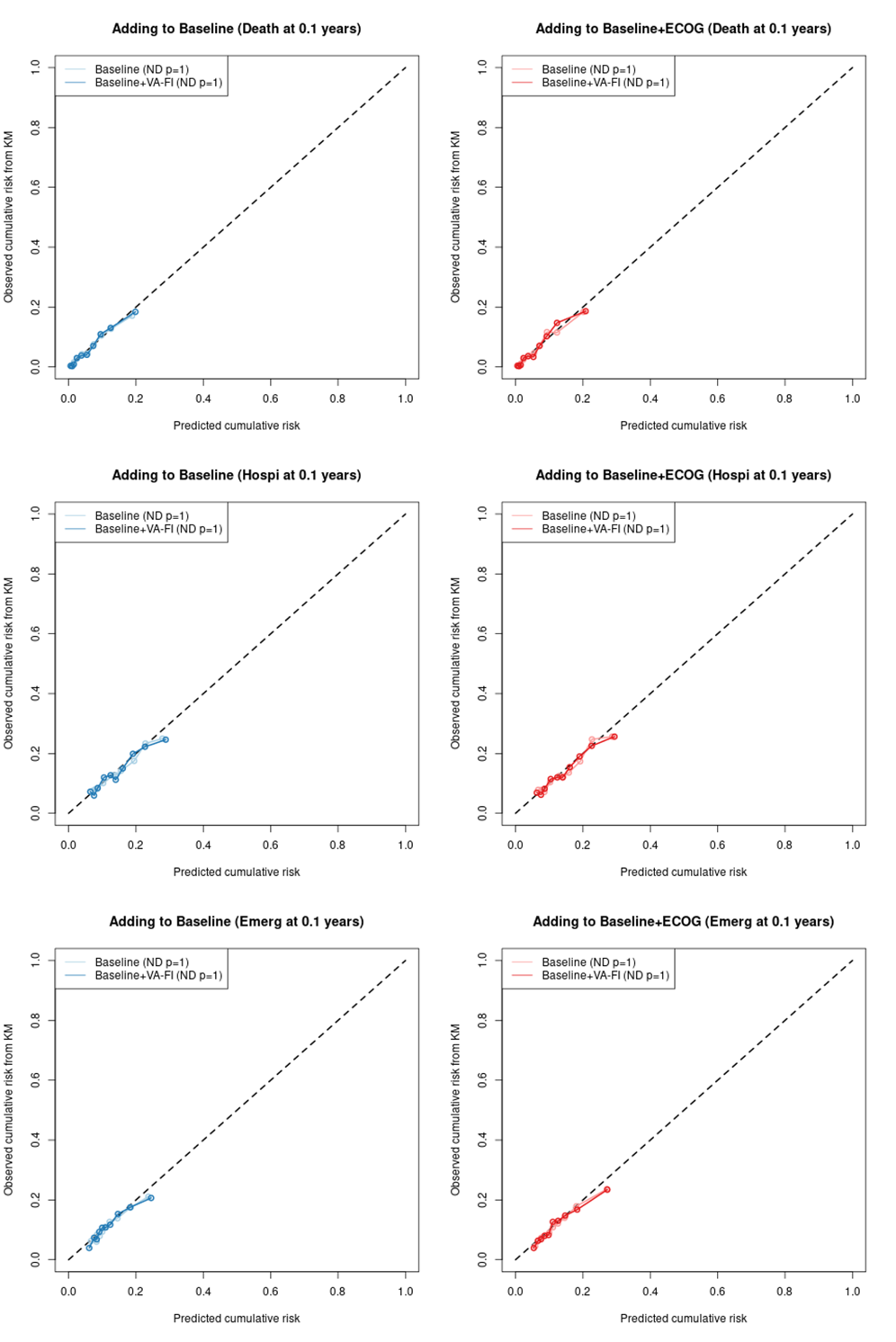


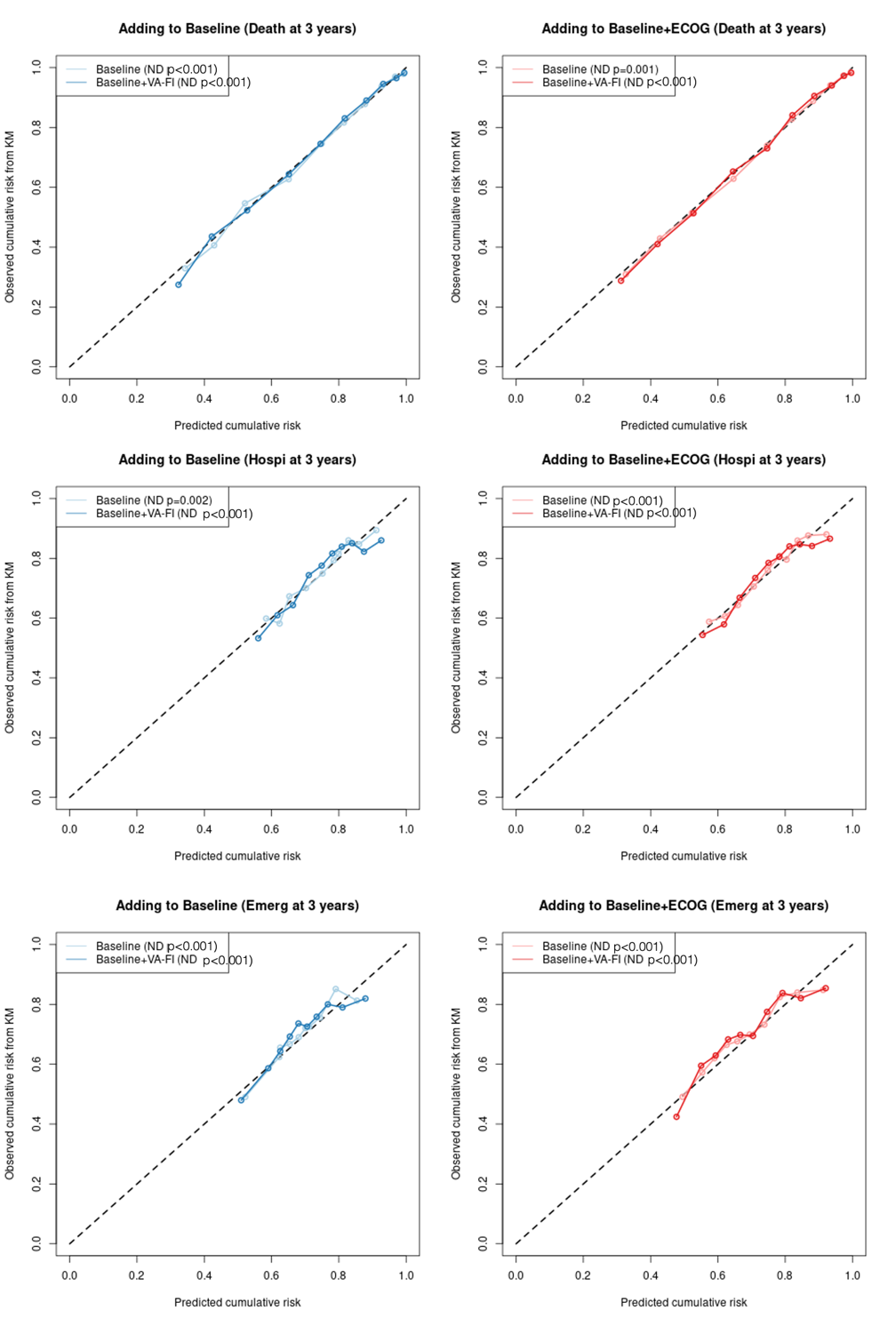


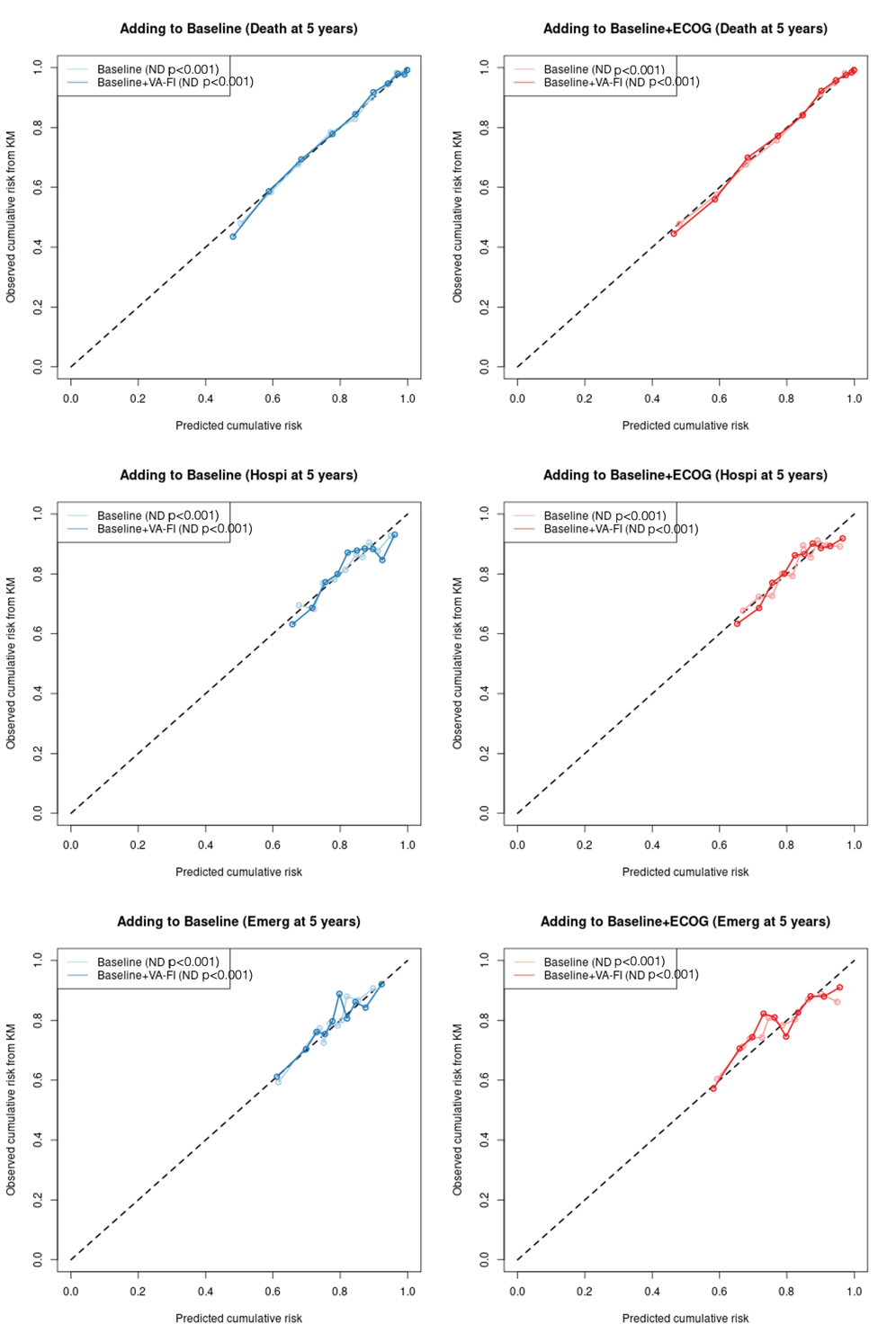


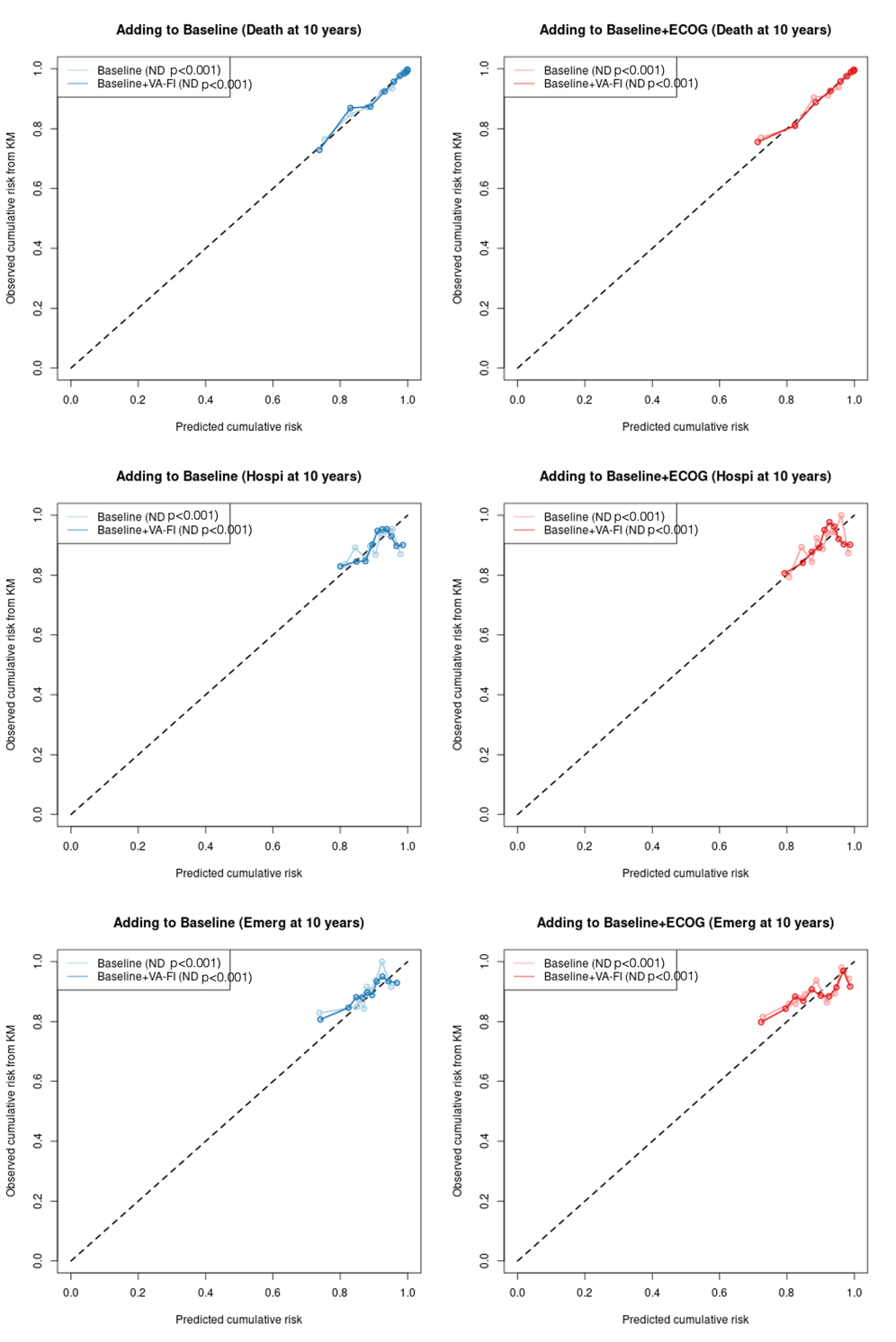


Figure S1: Calibration plot for predicted risk of mortality, hospitalization, and ER visit, by 1 month (0.1 year), 3 years, 5 years, and 10 years after diagnosis, based on Baseline and Baseline+VAFI models (Blue) and Baseline+ECOG and Baseline+ECOG+VAFI models (Red). The calibration plots show the observed risk, as estimated by Kaplan-Meier, against the mean predicted risk in deciles of the predicted risks. The p-values are from Nam-D’Agnostino tests, which test against the null that there are no differences between mean predicted and observed risks for each model. Abbreviations: ND, Nam-D’Agnostino test; VA-FI, VA Frailty Index; KM, Kaplan-Meier.
