# Supplementary material for: Prognostic value of the veterans affairs frailty index in older patients with non‐small cell lung cancer
Source: Cancer Med. 2022 Mar 26;11(15):3009–22. doi: 10.1002/cam4.4658 (PMC9359868; doi:10.1002/cam4.4658)
Supplement: Supplementary file 2 — Figure S2 [file CAM4-11-3009-s002.docx]

# 1-Year Calibration Plot among Patients with Known ECOG Status


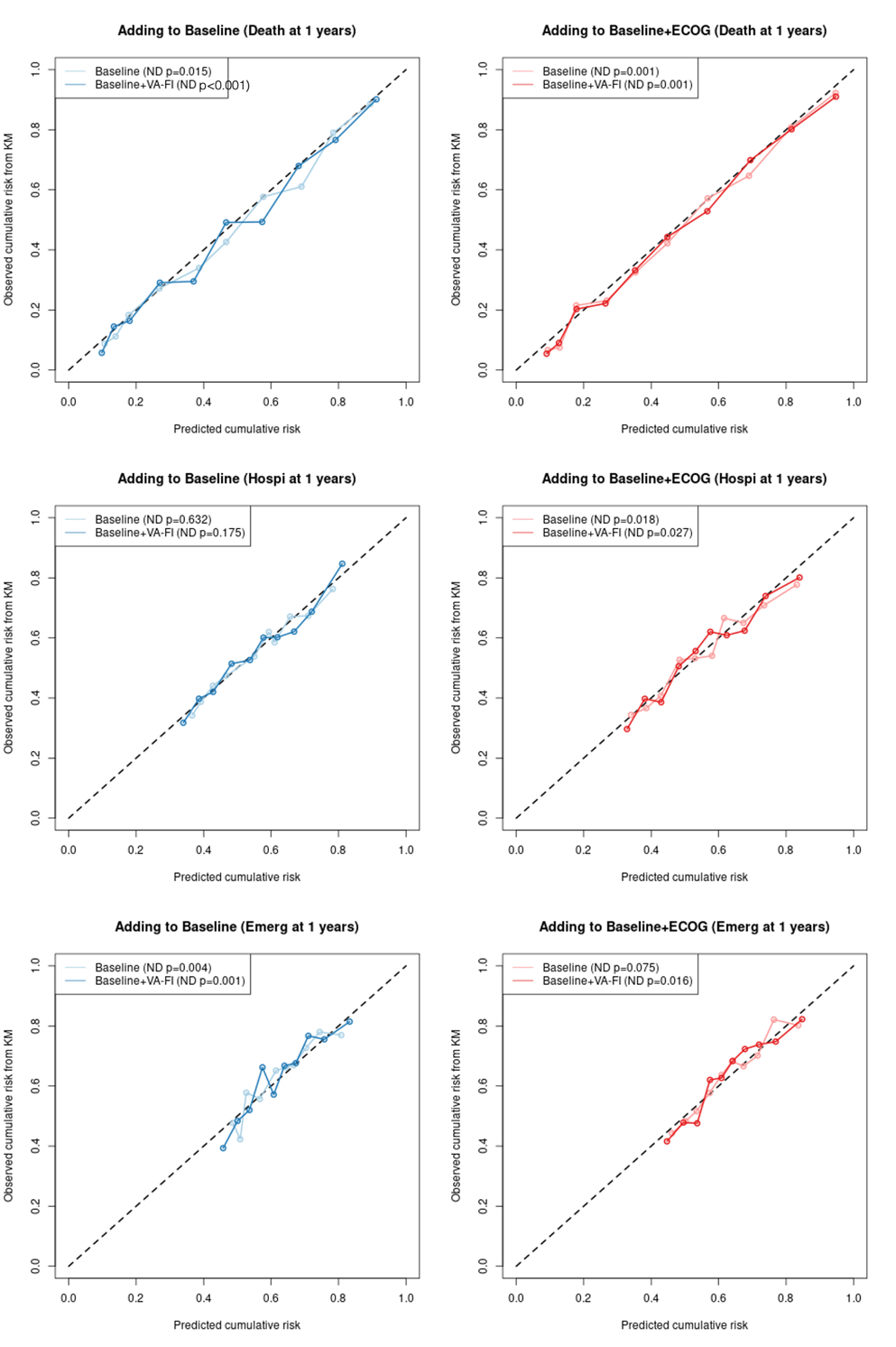


Figure S2: Calibration plot for predicted risk of mortality, hospitalization, and ER visit by 1 year after diagnosis, among those with known ECOG, based on Baseline and Baseline+VAFI models (Blue) and for Baseline+ECOG and Baseline+ECOG+VAFI models (Red). The calibration plots show the observed risk, as estimated by Kaplan-Meier, against the mean predicted risk in deciles of the predicted risks. The p-values are from Nam-D’Agnostino tests, which test against the null hypothesis that there are no differences between mean predicted and observed risks for each model. Abbreviations: ND, Nam-D’Agnostino test; VA-FI, VA Frailty Index; KM, Kaplan-Meier.
