# Supplementary material for: Prognostic value of the veterans affairs frailty index in older patients with non‐small cell lung cancer
Source: Cancer Med. 2022 Mar 26;11(15):3009–22. doi: 10.1002/cam4.4658 (PMC9359868; doi:10.1002/cam4.4658)
Supplement: Supplementary file 3 — Table S1 [file CAM4-11-3009-s001.docx]

# Time-Dependent AUC among Patients with Known ECOG Status

Table S1: Cumulative/dynamic time-dependent AUC and 95% CIs at landmark time points following diagnosis among patients with known ECOG status. Baseline refers to the baseline stratified Cox model for the hazard of each type of event using age, gender, and site of metastasis, stratifying by stage and histological subtype. “Baseline+ECOG” refers to the same model except that ECOG is added as a covariate, and similarly for “Baseline+VAFI” and “Baseline+ECOG+VAFI.” *p*-values are based on tests for the null that the AUC of each model differs from that of the “Baseline” model or the “Baseline+ECOG” model. For each landmark time and outcome, pairwise comparisons of the time-dependent AUCs are made with the Baseline (“vs baseline”) and Baseline+ECOG (“vs ecog”) models. Abbreviations: ECOG, Eastern Cooperative Oncology Group Performance Status; VA-FI, VA Frailty Index.
